# The Effects of Social Distance and Asymmetric Reward and Punishment on Individual Cooperative Behavior in Dilemma Situations

**DOI:** 10.3389/fpsyg.2022.816168

**Published:** 2022-04-19

**Authors:** Lei Zhang, Yan Jin, Lin Xia, Bibo Xu, Syed Mohamad Syed Abdullah

**Affiliations:** ^1^School of Educational Studies, Universiti Sains Malaysia (USM), Penang, Malaysia; ^2^School of Education Sciences, Huizhou University, Huizhou, China; ^3^Wuhan Fingu Electronic Technology Co., Ltd., Wuhan, China; ^4^Institute of Education, Hubei University, Wuhan, China

**Keywords:** social distance, asymmetric reward, asymmetric punishment, cooperative behavior, dilemma situations

## Abstract

The behavior decisions in social dilemmas are highlighted in sociological, economic, and social psychological studies. Across two studies, the iterated prisoner’s dilemma is used as a basic paradigm to explore the effects of social distance and asymmetric reward and punishment on an individual’s cooperative behavior. Experiment 1 (*N* = 80) used a 2 (social distance: intimacy vs. strangeness) × 2 (symmetry of rewards: symmetric rewards vs. asymmetric rewards) within-subject design and demonstrated that when there were only two options, namely, cooperation and defection, cooperative behavior was influenced by social distance and symmetry of rewards, respectively, and the interaction was not significant. Experiment 2 (*N* = 80) used a 2 (social distance: intimacy vs. strangeness) × 2 (symmetry of punishment: symmetric punishment vs. asymmetric punishment) within-subject design and showed that the cooperative behavior of participants decreased when the punishment option was added, and the two levels of symmetry and asymmetry were set. Specifically, compared with the symmetric punishment group, the asymmetric punishment group was more likely to choose a defection strategy and less likely to use a punishment strategy. Moreover, there was a marginal interaction effect between social distance and symmetry of punishment, and symmetry of punishment was a significant mediator in the relationship between social distance and individual cooperation. Specifically, asymmetric punishment reduced only the cooperation rate (CR) between participants and their friends. In conclusion, in dilemma situations, asymmetric reward did not influence individual cooperative behavior at different social distances, while asymmetric punishment did, because the sense of loss was more likely to awaken an individual’s social comparison motives.

## Introduction

When cooperation is better in their interests, why do both sides choose to defect? The issue has been receiving full attention in behavior, economics, and social psychology (Deck and Jahedi, 2015; Hilbe et al., 2015; Soutschek et al., 2015). The essence of this situation is described by the prisoner’s dilemma (Tucker, 1950). Two players have a choice between cooperation, C, and defection, D. If both players cooperate, they get more than if both defect, but defecting against a cooperator leads to the highest payoff while cooperating with a defector leads to the lowest payoff (Dreber et al., 2008).

As early, Akerlof (1997) advocated that a model of social distance could explain social decision-making or individual economic choices. He argued that social distance could measure the degree of intimacy in strategic interaction and had also proved to have a profound impact on individual choices (Akerlof, 1997). People’s willingness to cooperate with players often differs substantially with different social distances (Rachlin and Jones, 2008). Laboratory experimental evidence suggested that members of a team behave more altruistically when they perceive a closer social distance from other members of their team (Gee et al., 2020). This difference is due to the difficulty in maintaining long-term interactions between groups with different social distances and the possibility of obtaining long-term benefits from them (Bereczkei et al., 2007; Lamba and Mace, 2010; van Lange et al., 2011). The relationships between individuals and their friends or strangers can be regarded as different levels of social distance (Li et al., 2020). In laboratory studies and field trials, Binzel and Fehr (2013) found that individuals trust friends more than strangers. The study by Engelmann also found that individuals were more concerned about their inner group reputation than their outgroup reputation (Engelmann et al., 2013). In other words, the smaller the social distance, the higher the degree of reciprocity and the more cooperation (Zhang et al., 2017). However, this conclusion is based on the hypothesis that the two people are equal. What will a person do when he or she finds that he or she is playing the game with a friend in an asymmetrical situation? Will he or she still choose to cooperate? This is our study topic.

We are aware that the individuals’ unequal resource holdings affect the willingness to cooperate at a given time (Pansini et al., 2020). In view of the research results involving asymmetry, it is generally believed that asymmetry can have negative consequences for interpersonal relationships (Molho et al., 2019), which means asymmetry is typically associated with more selfish behavior (Handgraaf et al., 2008). We are aware that the individuals’ unequal resource holdings affect the willingness to cooperate at a given time (Hauser et al., 2019; Riccardo Pansini et al., 2020). In previous research, it was found that asymmetric settings would reduce the cooperative behavior of individuals, and they did not reach a consistent conclusion on the cooperative differences between the strong and weak individuals (Croson, 1999; Beckenkamp et al., 2007; Phillips, 2017). More specifically, some studies suggested that strong players were typically more cooperative than weak players (Bone et al., 2015). They believe that they have more “resources” or “abilities” and are obliged to contribute their resources to the group. However, some other studies have drawn different conclusion. Stouten et al. (2009) found that individuals who benefited more and less from public goods had no significant difference in their contributions to the group.

There are many different asymmetric situations, which generally include the following three types: (1) The asymmetry of benefits: the advantaged player gains more in the cooperative system than the disadvantaged player does; (2) The asymmetry of the interaction mode: the disadvantaged individuals either choose to pay a certain cost to participate in cooperation or adopt speculative strategies and defect when the advantaged individuals punish the uncooperative behavior of the disadvantaged players and reward cooperative behavior; and (3) The asymmetry of information: the so-called asymmetry of information is the different information owned by experimental participants, that is, the inconsistencies in the knowledge status between the cooperative receiver and the cooperative partner (Wu, 2014). In this study, we use two methods to construct the asymmetric benefit matrix: adjusting the benefits when both cooperate and adjusting asymmetric punishment.

It was generally posited in the previous study that in social interaction, people perceive and interpret the relationships between people from two basic perspectives: one is related to unity, intimacy, morality, passion, etc., and the other is related to ability, power, dominance, etc. (Liu and Hao, 2015). Therefore, it is important to ask this question: in social interactions, especially in social dilemmas, how do these two aspects affect people’s cooperative behaviors? To be more specific, social distance seems to represent the commonality of both players in the game, and the narrowing of social distance can promote the occurrence of cooperative behaviors (Lu et al., 2016). It seems that the commonality of both players in the game can effectively promote cooperation. However, the setting of an asymmetric social dilemma creates differences between the two players, which hinders cooperation to a certain extent.

To sum up, social distance and the asymmetry of the social dilemma have notable impacts on cooperative behaviors, and whether these two factors independently affect cooperative behaviors is an important question in this study.

## Study 1: The Effect of Social Distance and Asymmetric Reward on Cooperative Behaviors in the Prisoner’s Dilemma

z-Tree software was mainly used in this study to compile the computer program needed for the experiment. The program was mainly divided into three parts: the first part was the background information of the program, the second part was to let participants choose strategies, and the third part was to calculate their gains in one round and the total rewards based on the strategies chosen by the participants and to present the results to the participants. The participant could see his or her score after choosing every round.

### Participants

A total of 40 pairs of good friends were recruited in this study, including 20 male and 20 female pairs who were undergraduate or graduate students (*M*_*age*_ = 21.31, *SD* = 2.473) from Hubei University. They were right-handed and had normal vision or corrected visual acuity. Participants were recruited and asked to participate in the experiment together with a close friend of the same gender, and their relationships were repeatedly confirmed as good friends.

### Experimental Design and Material

This experiment adopted a 2 (social distance: intimate and strange) × 2 (symmetric and asymmetric rewards) within-subject design in which social distance was balanced, and the cooperation rate (CR) in social dilemmas was the main dependent variable in this experiment. A total of 20 pairs of men and 20 pairs of women were tested with either a close friend or a stranger first, while the rest were tested with a stranger first. The setting of the symmetry of rewards was reflected in the fact that when both chose cooperation at the same time, their rewards were different. The control group was presented with a matrix of symmetric rewards (as shown in Table 1). When both chose cooperation at the same time, their returns were the same, which were three points. The experimental group was presented with a matrix of asymmetric rewards (as shown in Tables 2, 3), and rewards for both sides were not equivalent when choosing cooperation, as shown in Table 2, which is presented from the perspective of the individual in a strong position in the asymmetric system, and Table 3, which is presented from the perspective of the individual in a weak position in the asymmetric system. Both parties in the game were in unequal or asymmetrical positions throughout this operation.

**TABLE 1:** Matrix of symmetric rewards.

		Opponent’s choice	
		A	B
**Your choice**	A	(3, 3)	(–5, 10)
	B	(10, –5)	(–3, –3)

**TABLE 2:** Asymmetric payoff matrix 1.

		Opponent’s choice	
		A	B
**Your choice**	A	(6, 3)	(–5, 10)
	B	(10, –5)	(–3, –3)

**TABLE 3:** Asymmetric payoff matrix 2.

		Opponent’s choice	
		A	B
**Your choice**	A	(3, 6)	(–5, 10)
	B	(10, –5)	(–3, –3)

### Experimental Program

All the subjects were tested in a specialized behavioral laboratory, which had four separate cubicles with a computer in each cubicle. The experiments were carried out in groups of four people consisting of two pairs of good friends or two pairs of good friends who were strangers to each other. Participants would be told in advance that he or she would play the games in a local area network with a friend first and then with a stranger or *vice versa*. The score of each round was determined jointly by the choices of both sides, and participants got the corresponding cash incentives according to the score they acquired in the game. Each person would have the same initial score of 300 points at the beginning of each game, which would increase or decrease according to the results of the subsequent game. The subjects should adopt strategies that were optimal for winning points from their standpoints.

After introducing the game rules to the subjects and confirming that the subjects had understood the rules, the four subjects were brought to the laboratory and informed that there would be no communication between the subjects. The subjects sat in a chair with their eyes being 1 m from the monitors. In the experiment, the guidelines would first appear on the computer screen, which was then followed by five trials for practice, and the game would officially start. The formal game stage contained two blocks: a block was a game with friends, and another block was with strangers. Each block of the game contained a total of 50 trials. Throughout the whole experiment, feedback was not provided until the end of the experiment, when the final participants would be presented with the total score of each individual and the exchange of money.

### Results

In this experiment, there were a total of 80 subjects, and the cooperation rate for each variable is shown in Table 4.

**TABLE 4:** Descriptive statistics for each variable.

	Variable (V)	Number (N)	Cooperation rate (CR)
Gender	male	40	0.743 ± 0.244
	female	40	0.778 ± 0.198
Social distance	strangeness	80	0.675 ± 0.29
	intimacy	80	0.845 ± 0.217
Symmetry of rewards	symmetry	40	0.857 ± 0.154
	asymmetry	40	0.664 ± 0.238
Asymmetrical position	the strong	20	0.69 ± 0.239
	the weak	20	0.638 ± 0.228

A 2 (gender: male, female) × 2 (social distance: intimacy, strangeness) × 2 (reward symmetry: reward asymmetry) repeated measures analysis of variance (ANOVA) was performed for the cooperation rate. The repeated measures ANOVA results showed that the main effect of gender was not significant, with *F*_(1,77)_ = 0.616, *P* = 0.435 > 0.01, η*_*P*_*^2^ = 0.008, and observed power = 0.121, indicating that there was no significant difference between the cooperation rate of men (0.743) and that of women (0.778) in the experiment. The main effect of social distance was significant, with *F*_(1,77)_ = 34.402, *P* < 0.001, η*_*P*_*^2^ = 0.309, and observed power = 1.000, which indicated that the cooperation rate (0.845) for the experiment with friends was significantly higher than that for the experiment with strangers (0.675). The main effect of reward symmetry was significant, with *F*_(1,77)_ = 18.413, *P* < 0.001, η*_*P*_*^2^ = 0.193, and observed power = 0.989. However, the interaction effects of gender × social distance, gender × rewards symmetry, social distance × rewards symmetry, and gender × social distance × rewards symmetry were not significant (*P* > 0.2).

A simple effect analysis was then conducted. After an independent sample *t*-test was carried out for the data with the asymmetry of rewards, the results were as follows: *F* = 1.138, *P* = 0.293 > 0.01, which showed homogeneity of variance; *t* = 0.694, *P* > 0.01, which showed that there was no significant difference in the cooperation rate between the strong side of the income asymmetry and the weak side.

### Discussion

When there were two options: cooperation and defection, the main effect of social distance was significant, which is consistent with the previous findings of Lu et al. (2016). The cooperation rate of the reward symmetry group is significantly higher than that of the reward asymmetry group. This is similar to many researchers’ conclusion (Sheposh and Gallo, 1973). The rewards are set in an asymmetric distribution pattern, namely, that if the players choose the cooperation strategy, the gains of two sides will be unequal, manually placing two individuals in asymmetric positions: one is the strong side, one is the weak, and other settings are identical to the original prisoner’s dilemma (Croson, 1999). When they both choose to cooperate, the benefits are the greatest for the group, but individually, one side gains more than the other. If one person chooses to cooperate and one person chooses to defect, the defector earns and the cooperator loses. Both sides lose when they choose to defect. In this case, there was a significant difference in the cooperation rate between symmetric and asymmetric groups, that is, the asymmetric setting of rewards urges individuals to choose the strategy of non-cooperation, which seems safer. Wang et al. (2010) found that the frequency of the conflict between the two sides of the game is closely related to the comparison of the strength of the two sides of the game by using the “Eagle pigeon game” model and game theory. Sheposh and Gallo (1973) explain this in a social dilemma, where participants are not more concerned with the absolute benefits of the individual, but the relative benefits of each other. The reason why the disadvantaged participants adopt uncooperative behavior is to reduce the actual income of their opponents, while the dominant participants pay more attention to the unbalanced compensation brought about by the asymmetric structure than the promotion of their own income.

The main reason for the results in this study is that asymmetric income stimulates the individual’s inner consciousness of “fairness.” Although everyone will pursue “benefit maximization” in theory, individuals can clearly see their own and each other’s scores in each round of experiments. Thus, although the participants were aware that the greatest benefit comes only with cooperation, there were imparities in the incomes of individuals, which means that the incomes were different for finishing the same task. This is contrary to our daily concept of “fairness,” or violates the principle of “fairness maximization.” Therefore, in order to maintain this concept of fairness, individuals tend to choose the option of non-cooperation compared with the symmetric group.

## Experiment 2: The Effect of Social Distance and Asymmetric Punishment on Cooperative Behavior in the Prisoner’s Dilemma

### Participants

In this experiment, 40 pairs of good friends were recruited, including 20 male and 20 female pairs who were undergraduate or graduate students. The age distribution was *M* = 20.14 years, *SD* = 1.881. The participants of each pair in the experiment were of the same sex, and they all were good friends (confirmed by the lead experimenter). Among them, half were male, and all were right-handed and had normal vision or corrected vision.

### Experimental Design and Experimental Materials

In this study, a 2 (social distance: intimacy and strangeness) × 2 (symmetrical punishment and asymmetrical punishment) within-subject design was adopted. Social distance was balanced between subjects. A total of 20 male and 20 female pairs participated in the experiment with close friends first, while the rest of the subjects participated in the experiment with strangers first. In this experiment, compared with Experiment 1, punishment (C) was added to their choices, besides cooperation (A) and defection (B).

In order to explore the effect of asymmetric punishment on cooperative behavior, a control group and an experimental group were set up. In the control group, a symmetric punishment matrix was presented (as shown in Table 5), that is, when the participants chose punishment, they both lost 1 unit, while their opponents lost 2 units; the experimental group was presented with the asymmetric reward matrix (as shown in Tables 5, 6), namely, when the participants chose to punish, one lost one unit and the other lost two units, while the other lost one unit and their opponent lost four units. Table 5 is presented from the perspective of an individual in a strong position in the asymmetric system, and Table 7 is presented from the perspective of an individual in a weak position in the asymmetric system. Through this operation, both parties in the game were in unequal or asymmetrical positions.

**TABLE 5:** Matrix 1 of asymmetric punishments.

		Opponent’s choice		
		A	B	C
Your choice	A	(1, 1)	(–2, 3)	(–3, 1)
	B	(3, –2)	(0, 0)	(–1, –2)
	C	(1, –5)	(–2, –3)	(–3, –5)

**TABLE 6:** Matrix of symmetric punishments.

		Opponent’s choice		
		A	B	C
Your choice	A	(1, 1)	(–2, 3)	(–3, 1)
	B	(3, –2)	(0, 0)	(–1, –2)
	C	(1, –3)	(–2, –1)	(–3, –3)

**TABLE 7:** Matrix 2 of asymmetric punishments.

		Opponent’s choice		
		A	B	C
Your choice	A	(1, 1)	(–2, 3)	(–5, 1)
	B	(3, –2)	(0, 0)	(–1, –2)
	C	(1, –3)	(–2, –1)	(–5, –3)

### Experimental Procedure

The procedure was same as Experiment 1.

### Results

#### Description of Statistical Results

In this experiment, there were a total of 80 participants, and the cooperation rate, defection rate (DR), and punishment rate (PR) of each variable are shown in Table 8 (in this study, the defection rate was also called the non-cooperation rate).

**TABLE 8:** Descriptive statistics for each variable.

	Variables (V)	Number (N)	Cooperation rate (CR)	Defection rate (DR)	Punishment rate (PR)
**Gender**	Male	40	0.570 ± 0.273	0.374 ± 0.246	0.057 ± 0.076
	female	40	0.556 ± 0.273	0.424 ± 0.274	0.095 ± 0.150
Social distance	strangeness	80	0.414 ± 0.351	0.543 ± 0.351	0.117 ± 0.230
	intimacy	80	0.712 ± 0.352	0.304 ± 0.358	0.085 ± 0.220
Symmetry of punishments	symmetry	40	0.626 ± 0.249	0.349 ± 0.251	0.099 ± 0.158
	asymmetry	40	0.500 ± 0.280	0.448 ± 0.262	0.052 ± 0.055
Asymmetry of position	the strong	20	0.520 ± 0.261	0.424 ± 0.253	0.057 ± 0.064
	the weak	20	0.480 ± 0.304	0.473 ± 0.275	0.048 ± 0.046

#### Results of Analysis of Variance

##### Repeated Measures Analysis of Variance of 2 (Gender: Male, Female) × 2 (Social Distance: Itimacy, Strangeness) × 2 (Symmetry of Punishment: Symmetric Punishment, Asymmetric Punishment) Performed for Cooperation Rate

The results of the repeated measures ANOVA showed that the main effect of gender was not significant, with *F*_(1,77)_ = 0.053, *P* = 0.818 > 0.01, η*_*p*_*^2^ = 0.001, and observed power = 0.056, indicating that there was no significant difference between the cooperation rate of men (0.570) and that of women (0.556).

The main effect of social distance was significant, with *F*_(1,77)_ = 36.043, *P* < 0.001, η*_*p*_*^2^ = 0.319, and observed power = 1.000, which indicated that the cooperation rate (0.712) of the experiment with friends was significantly higher than that of the experiment with strangers (0.414).

**FIGURE 1:**
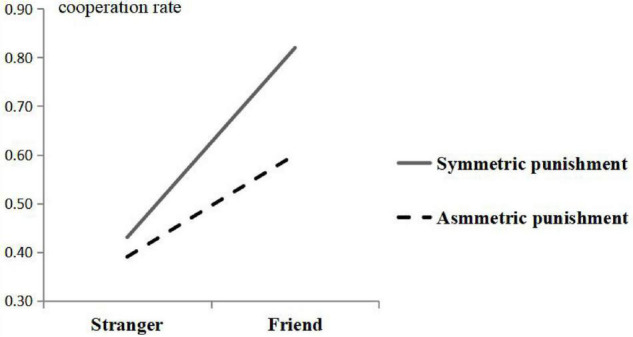
Interaction effect of social distance and symmetry of punishment.

The main effect of punishment symmetry was significant, with *F*_(1,77)_ = 4.508, *P* = 0.037 < 0.05, η*_*p*_*^2^ = 0.055, and observed power = 0.554. The interaction effect of social distance and punishment symmetry was nearly significant, with *F*_(1,77)_ = 3.105, *P* = 0.082 < 0.1, η_*p*_^2^ = 0.039, and observed power = 0.413, as shown in Figure 1. However, the interaction effects of gender × social distance, gender × punishment symmetry, and gender × social distance × punishment symmetry were not significant (*P* > 0.2).

Then, a simple effect analysis was conducted. After an independent sample *t*-test was carried out for the data with the asymmetry punishment, the results were as follows: *F* = 0.781, *P* = 0.382 > 0.01, which showed homogeneity of variance; *t* = 0.446, *P* > 0.01, which showed that there was no significant difference in the cooperation rate between the strong side of the punishment asymmetry and the weak side.

#### Repeat Measurement Analysis of Variance of 2 (Gender: Male, Female) × 2 (Social Distance: Itimacy, Strangeness) × 2 (Symmetry of Punishments: Symmetric Punishment, Asymmetric Punishment) Performed for Defection Rate

The results of repeated measures ANOVA showed that the main effect of gender was nearly significant, with *F*_(1,77)_ = 3.784, *P* = 0.055, η*_*p*_*^2^ = 0.047, and observed power = 0.384, indicating that the non-cooperation rate of men (0.374) was slightly lower than that of women (0.424).

The main effect of social distance was significant, with *F*_(1,77)_ = 24.118, *P* < 0.001, η*_*p*_*^2^ = 0.239, and observed power = 0.998, which indicated that the rate of defection (0.543) in the experiment with strangers was significantly higher than that of defection (0.304) in the experiment with friends.

The main effect of the symmetry of punishment was significant, with *F*_(1,77)_ = 23.553, *P* < 0.001, η*_*p*_*^2^ = 0.234, and observed power = 0.998. However, the interaction effects of gender × social distance, gender × punishment symmetry, social distance × punishment symmetry, and gender × social distance × punishment symmetry were not significant (*P* > 0.2).

Then, an independent sample *t*-test was carried out for the defection rate of the punishment asymmetry, *F* = 0.040, *P* = 0.842 > 0.01, homogeneity of variance was shown, *t* = –1.735, *P* > 0.01, indicating that there was no significant difference in the defection rate between the strong side of the punishment asymmetry and the weak side of the punishment asymmetry.

#### Repeated Measures Analysis of Variance of 2 (Gender: Male, Female) × 2 (Social Distance: Itimacy, Strangeness) × 2 (Punishment Symmetry: Symmetry, Asymmetry) Performed for Punishment Rate

The results of repeated measures ANOVA showed that the main effect of gender was significant, with *F*_(1,77)_ = 7.200, *P* = 0.009 < 0.01, η*_*P*_*^2^ = 0.086, and observed power = 0.755, indicating that there was a significant difference between the punishment rates displayed by men and women in the experiment.

The main effect of social distance was not significant, with *F*_(1,77)_ = 0.825, *P* = 0.366 > 0.05, η*_*P*_*^2^ = 0.011, and observed power = 0.146, which indicated that there was no significant difference between the punishment rate of the experiment with friends (0.084) and that of the experiment with strangers (0.117).

The main effect of the symmetry of punishment was significant, with *F*_(1,77)_ = 9.864, *P* = 0.002 < 0.05, η*_*p*_*^2^ = 0.114, and observed power = 0.873. Compared with the symmetric punishment group, the asymmetric punishment group had more punishment behaviors.

**FIGURE 2:**
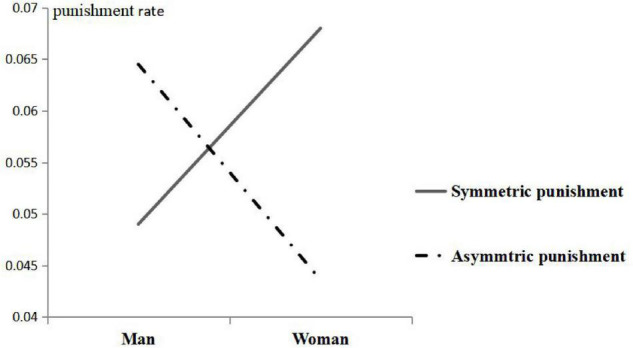
Interaction of gender and symmetry of punishment.

The interaction effect of gender × punishment symmetry was significant, with *F*_(1,77)_ = 7.742, *P* = 0.007 < 0.05, η*_*P*_*^2^ = 0.092, and observed power = 0.784, as shown in Figure 2. However, the interaction effects of gender × social distance, gender × punishment symmetry, social distance × punishment symmetry, and gender × social distance × symmetry of punishment were not significant (*P* > 0.2).

Then, an independent sample *t*-test was carried out for the punishment rate of asymmetry of punishment, and the results were as follows: *F* = 0.290, *P* = 0.593 > 0.01, which showed homogeneity of variance; *t* = 0.484, *P* > 0.01, indicating that there was no significant difference in the punishment rate between the strong side of asymmetrical punishment and the weak side of asymmetric punishment.

#### Comparison With the Results of Experiment 1

An important difference between Experiment 1 and Experiment 2 was that the option of “punishment” was added in Experiment 2, so the differences in the experimental results of the control group in the next two experiments could be compared (as shown in Table 9), and it was found that adding the punishment option reduced the cooperative behavior of the participants.

**TABLE 9:** Comparison of Experiment 1 and Experiment 2.

Variables (V)		Experiment 1	Experiment 2
Sex	male	40	40
	female	40	40
Social distance	strangeness	0.675 ± 0.29	0.414 ± 0.351
	intimacy	0.845 ± 0.217	0.712 ± 0.352
Selection strategy		cooperation, competition	Cooperation, competition, punishment
Cooperation rate		0.877 ± 0.248	0.614 ± 0.254
Defeat rate		0.133 ± 0.248	0.328 ± 0.226
Punishment rate		—	0.059 ± 0.078

### Discussion

#### The Influences of Social Distance and Asymmetric Punishment on Individual’s Cooperative Behavior

In both cases where the cooperation rate or the defection rate was taken as the dependent variable, it was found that the main effects of social distance and punishment symmetry were significant. The punishment setting made the punishment power of both sides unequal: one was the strong side of the sanction and the other was the weak side, which would cause psychological changes in the subjects. The results showed that the cooperation rate of the asymmetric group was lower than that of the symmetric group, the defection rate of the asymmetric group was higher than that of the symmetric group, and the differences between them were statistically significant. Therefore, asymmetric groups were more inclined to choose a defection strategy to seek self-protection and achieve the purpose of protecting themselves. The study conducted by Nikiforakis et al. (2010) also yielded similar results: not only did the setting of asymmetric penalties not promote cooperation, but it also hindered cooperation. In this study, punishment and cooperation were both risky behaviors, so the asymmetric settings made individuals more inclined to choose conservative behaviors.

There was a marginally significant interaction effect on cooperative behavior between social distance and punishment symmetry. As shown in Figure 1, there was no significant difference in the cooperation rate between the subjects with strangers, whether in the symmetric group or the asymmetric group, and the setting of asymmetric punishment mainly reduced the cooperation rate between the subjects with good friends in the game. This phenomenon is very common in life: a beggar will not envy a millionaire but will envy a beggar with a higher income than his. If the Chinese people often compare themselves with African refugees, then the Chinese people will certainly have no complaints, and everyone will be happy, but unfortunately, we will not make such comparisons, because they are meaningless, that is, people always tend to compare themselves with individuals of the same class. Festinger (1954) put forward “the social comparison theory” in his study that individuals use others as a measure of comparison in the absence of objectivity. According to Self-Evaluation Maintenance Model (SEM), comparison occurs when the actor is outperformed by a close other on a relevant dimension. In other words, in our study, when an individual found his or her friend had a more powerful punishment ability, it would motivate the individual’s desire for comparison, which made the comparer to react in some way to the existence of the difference with a behavior change (Gerber et al., 2018). So the comparer in the asymmetric punishment group was less cooperative than that of the symmetric punishment group when playing the game with friends.

The main effect of gender on non-cooperative behavior was significant, that is, women chose non-cooperative strategies more than men, which is consistent with the finding of a study by Wu (2014). For one thing, there might be reasons for this result, which were rooted in the Eastern culture. In Eastern countries, women have always been in a relatively disadvantaged position, thus leading women to avoid risks and choose to “protect themselves.” On contrary, this can be explained by the gender schema theory (Spence, 1993). It is generally believed that boys should be independent, adventurous, motivated, and competitive, with high motivation for achievement, while girls are highly dependent, have strong security needs, cooperate and pursue interpersonal harmony, and have strong interpersonal skills and sensitivity to interpersonal relationships. Therefore, women are relatively more conservative. Cooperation is relatively risky because they each risk their own points. Thus, compared with men, women will choose defection more.

#### The Influence of Social Distance and Asymmetric Punishment on Individual Punishment Rate

First, let’s explore why “punishment” exists. Yamagishi’s (1992) research showed that when members of a group expected less cooperation, people tended to set up or introduce some kind of punishment system, and punishment came from an individual’s emotional needs. Fehr and Gachter (2002) also showed that individuals tended to punish individuals who contributed less to the group, which meant punishment options were introduced to increase cooperation rates. By comparing the results of Experiment 1 and that of Experiment 2 in this study, we found that the rate of cooperation became significantly lower after a penalty was added, which means punishment reduced people’s cooperative behavior. Mulder (2006) showed that punishment undermines trust and will make the cooperation motive change from being internal to external. The research done by Tenbrunsel and Messick (1999) showed that punishment systems had a negative impact on the decision-making framework used by individuals in a social dilemma. It made individuals consider their behavior as a market transaction, an economic act, and forget the moral dimension behind this behavior, thus reducing the cooperation rate.

In this study, the main effect of gender on punitive behavior was significant: women used punishment more than men. The original purpose of the punishment strategy was to sanction those who did not cooperate so that they would abandon the principle of self-interest and choose to cooperate, that is, to sacrifice one’s own interests for the benefit of the group. After all, when individuals chose to punish, he or she would lose his or her own profits. So in this respect, it was also an act of altruistic punishment. Similar to the results of this study, Botelho et al. (2000) used the Americans and Russians as subjects, and male respondents from both countries were found to use less altruistic punishment than women. According to the theory of differential socialization theory, women are socially more ethical than men, conduct more reflection and assessment on their own behavior, and are also more concerned about whether their behavior will have a negative impact on others. On contrary, the socialization of men makes them more individualistic. Compared to other people’s or collective interests, they are more concerned with their own interests. Therefore, in the whole study, women’s motivation to uphold social norms had always prevailed. As a result, there was an increase in altruistic punishment (Whitley et al., 1999). In addition, people tend to give individuals who make altruistic punishments greater social credibility since they defend fairness at their own expense. From this point of view, altruistic punishment brings both temporary losses and long-term benefits, and this is consistent with the finding that women are more likely than men to delay gratification (Silverman, 2003).

The main effect of punishment symmetry was significant, that is, the asymmetric group used the punishment strategy more than the symmetric group did. In this study, asymmetric group subjects were told that the setting of the options for the next experiment would be different before the experiment began, and through the previous rounds of experiments, both sides would soon realize who was strong and who was weak, which would activate an individual’s “sense of fairness” and “fair emotion,” thus affecting individual decision-making. Compared with symmetric groups, non-symmetric groups used non-cooperative strategies more, so this would stimulate asymmetric group individuals to use more punishment to maintain “justice.”

Gender and punishment symmetry had a significant interaction effect on the punishment rate. In the symmetric group, the punishment rate was significantly higher in women than in men, and the reason was as seen above (the discussion in the main effect of gender). In the asymmetric group, men’s punishment rate is significantly higher than that of women, and this might be because women tend to be cognitively conservative; costly punishment option is essentially a risky behavior because its essence is loss, and asymmetric setting makes women deepen its risk cognition, so women still tend to choose a conservative strategy: defection.

## General Discussion

Through the above analysis, it was found that social distance and reward symmetry independently affect cooperative behavior, while social distance and punishment symmetry interact with cooperative behavior. Cooperation and defection correspond to the collective and individual (i.e., benefit orientation) of the rational system, and when asymmetric returns are set, it does not affect the individual’s decisions at different social distances, which means the individual cooperates more with friends. Punishment is caused by emotional factors, so it represents the emotional system. When the symmetry of punishment is set, it stimulates the individual’s social perception (cognition) of social distance, especially for the change of the social perception of the different social distances, that is, the cooperation rate between good friends is reduced, mainly because of symmetric punishment or asymmetric punishment. Punishment options can lead to the loss of a unit, so this subjective “sense of loss” awakens the individual’s social comparative motivation, thus affecting the individual’s perception of social distance.

Therefore, when the social perception influencing factors (horizontal direction) representing unity and morality and the social perception influencing factors (vertical direction) representing strength and power act on the individual, their modes of action are not always the same. It is possible for them to act independently or interact with each other, which is related to the vertical setting. A study by Tan et al. (2017) showed that in low-risk situations, people consider only the ability of the game object, but in high-risk situations, people will consider the game object’s ability and social distance at the same time to increase the possibility of profit. A study has proved that in similar game scenarios, the nature of the situation itself will affect the interpretation of the task and then change the game tendency (Duffy and Kornienko, 2010; Bosco, 2012), and the addition of punishment options in this study will change the individual’s perception of the game situation. Therefore, punishment symmetry has a mediating effect on the role of social distance on individual cooperative behavior.

## Conclusion

By using the repeated prisoner’s dilemma paradigm on z-Tree software and exploring the influences of social distance and asymmetric reward and punishment on individual decision-making behaviors in social dilemmas, it was found that (1) Adding the punishment option will reduce individual cooperative behavior; (2) Social distance, reward symmetry, and punishment symmetry promote cooperative behavior to some extent; (3) The punishment symmetry mediates the effect of social distance on individual cooperative behavior; (4) Compared to the symmetric punishment groups, asymmetric punishment groups tend to use defection strategies and use less punishment strategies; (5) Gender affects both defection and punishment, that is, compared with men, women will choose more defection strategies and punishment strategies; and (6) Gender and punishment symmetry have a significant interaction effect on punishment behavior.

## Data Availability Statement

The raw data supporting the conclusions of this article will be made available by the authors, without undue reservation.

## Ethics Statement

The studies involving human participants were reviewed and approved by Institute of Education of Hubei University. The participants provided their written informed consent to participate in this study.

## Author Contributions

All authors helped in writing the manuscript, contributed to the article, and approved the submitted version.

## Conflict of Interest

LX was employed by company Wuhan Fingu Electronic Technology Co., Ltd. The remaining authors declare that the research was conducted in the absence of any commercial or financial relationships that could be construed as a potential conflict of interest.

## Publisher’s Note

All claims expressed in this article are solely those of the authors and do not necessarily represent those of their affiliated organizations, or those of the publisher, the editors and the reviewers. Any product that may be evaluated in this article, or claim that may be made by its manufacturer, is not guaranteed or endorsed by the publisher.
